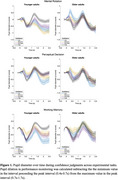# Investigating the role of LC‐NA system activity in age differences in performance monitoring: insights from pupillometry

**DOI:** 10.1002/alz70861_108829

**Published:** 2025-12-23

**Authors:** Sabrina Lenzoni, Joshua Horngacher, Jamie Kofler, Philipp Feistimantl, Ophir Gat, Dorothea Hämmerer

**Affiliations:** ^1^ University of Innsbruck, Innsbruck, Tyrol Austria; ^2^ Institute of Cognitive Neuroscience, University College London, London UK; ^3^ Wellcome Centre for Human Neuroimaging, Queen Square Institute of Neurology, University College London, London UK; ^4^ Institute of Cognitive Neurology and Dementia Research (IKND), Otto‐von‐Guericke University, Magdeburg Germany; ^5^ Center for Behavioral Brain Sciences, Magdeburg Germany; ^6^ German Center for Neurodegenerative Diseases, Magdeburg Germany; ^7^ Wellcome Centre for Human Neuroimaging, University College London (UCL), Queen Square Institute of Neurology, London UK; ^8^ Institute of Cognitive Neurology and Dementia Research, Otto‐von‐Guericke University Magdeburg, Magdeburg Germany

## Abstract

**Background:**

Performance monitoring is pivotal for learning and decision‐making across the lifespan. While evidence suggests that these abilities decline with age and older individuals often tend to overestimate their abilities, that is, show imprecise metacognitive evaluations of their performance. Recent evidence indicates that increased activity of the locus coeruleus‐noradrenaline (LC‐NA) system may subserve performance monitoring processes. The locus coeruleus (LC), which plays a critical role in various cognitive functions, is known to undergo structural and functional decline with age. This study aimed to investigate whether changes in pupil size—an indirect and non‐exclusive distal measure of LC activation—are associated with performance appraisal impairments in aging.

**Method:**

40 healthy younger adults (20‐30 y.o.) and 27 healthy older adults (60‐75 y.o.) completed working memory, perceptual decisions and mental rotation tasks while providing trial‐by‐trial confidence judgments during eye‐tracking recordings.

**Result:**

Results indicated that older adults made more errors, exhibited slower responses and were less confident about performance than younger adults across all tasks. In both age groups, higher confidence in performance accuracy was associated with greater accuracy and faster responses, suggesting that confidence judgments were well‐aligned with behavioral performance across groups. Notably, pupil size was larger during higher confidence trials for both groups but was consistently larger in older adults across all tasks

**Conclusion:**

Our findings suggest that pupil dilation serves as a reliable marker of performance monitoring across cognitive domains in different age groups. They do not support the notion that performance monitoring declines with age. Furthermore, our findings align with the notion that the LC‐NA system may become overactive in aging, potentially compensating for neuronal loss to sustain cognitive function.